# Somatic loss of *WWOX* is associated with *TP53* perturbation in basal-like breast cancer

**DOI:** 10.1038/s41419-018-0896-z

**Published:** 2018-08-06

**Authors:** Suhaib K. Abdeen, Uri Ben-David, Aya Shweiki, Bella Maly, Rami I. Aqeilan

**Affiliations:** 1 Lautenberg Center for Immunology and Cancer Research, IMRIC, Hebrew University-Hadassah Medical School, IMRIC, Jerusalem, Israel; 2 Cancer Program, Broad Institute of Harvard and MIT, Cambridge, MA USA; 3 Department of Pathology, Hadassah University Hospital, Jerusalem, Israel; 4 Department of Cancer Biology and Genetics, The Wexner Medical Center, The Ohio State University, Columbus, OH USA

## Abstract

Inactivation of WW domain-containing oxidoreductase (*WWOX*), the gene product of the common fragile site FRA16D, is a common event in breast cancer and is associated with worse prognosis of triple-negative breast cancer (TNBC) and basal-like breast cancer (BLBC). Despite recent progress, the role of WWOX in driving breast carcinogenesis remains unknown. Here we report that ablation of *Wwox* in mammary tumor-susceptible mice results in increased tumorigenesis, and that the resultant tumors resemble human BLBC. Interestingly, copy number loss of *Trp53* and downregulation of its transcript levels were observed in the *Wwox* knockout tumors. Moreover, tumors isolated from *Wwox* and *Trp53* mutant mice were indistinguishable histologically and transcriptionally. Finally, we find that deletion of *TP53* and *WWOX* co-occurred and is associated with poor survival of breast cancer patients. Altogether, our data uncover an essential role for WWOX as a *bona fide* breast cancer tumor suppressor through the maintenance of p53 stability.

## Introduction

Breast cancer is the most common malignancy in women and second to lung carcinoma in cancer mortality^1^. One of the greatest advances in the last few years has been the molecular categorization of breast cancer based on gene expression profiles. Transcriptomic analyses of human breast tumors have led to classification of several molecular subtypes with distinctive gene profiles and clinical relevance^2–4^. These molecular subtypes are strongly associated with survival outcome, with the basal-like subtype (BLBC) having the worst prognosis^2–4^. Identification of new molecular targets and modeling of BLBC would hence greatly enhance our understanding of this aggressive subtype and aid in better management.

The WW domain-containing oxidoreductase (*WWOX*) gene spans one of the most active common fragile sites in the human genome located at the long arm of chromosome 16: FRA16D^5,6^. WWOX is commonly altered in breast cancer^7–9^. In particular, it has been shown that WWOX protein levels are reduced or absent in triple-negative breast cancer (TNBC) and in BLBC^10–15^. Beside genomic rearrangements, hypermethylation of the regulatory region of WWOX has been documented in neoplastic but not in paired adjacent non-neoplastic tissues^16,17^. Importantly, restoration of WWOX expression inhibits breast cancer cell growth both in vitro and in vivo, further proposing a tumor suppressive function^17^. These observations led us to question whether WWOX possesses a driver role in tumor suppression in genetically engineered mouse models.

Existing evidence using animal models has indeed linked WWOX with tumor suppressive functions^18–21^. Modeling WWOX loss in drosophila revealed that WWOX expression is required for efficient removal of tumorigenic cells via TNFα/Egr-mediated cell death, which was shown to be dependent on caspase-3 activity^22^. Furthermore, a number of *Wwox* mutant mouse models have also suggested tumor suppressive roles for WWOX. In particular, aged germline *Wwox*-heterozygous mice on mixed background developed higher incidence of spontaneous lung tumors and B-cell lymphomas^23,24^, and those on C3H genetic background developed mammary tumors with 50% penetrance^25^. Some of these tumors had retained the other wild type allele, suggesting haploinsufficiency of WWOX function^23,25^. Importantly, mammary tumors in *Wwox*-heterozygous C3H mice were mostly ER-negative and PR-negative, expressing CK-14, hence reminiscent of the common WWOX inactivation in TNBC and in particular BLBC^25^. Despite these lines of evidence, however, no proof was established linking somatic loss of WWOX in mammary epithelium with mammary tumor advantage.

Several studies have shown that the WWOX protein antagonizes tumorigenesis by promoting apoptosis^26,27^, rewiring metabolism^28,29^ and more recently, maintaining genome integrity^30,31^. WWOX, mainly through its first WW domain^32,33^, interacts with partner proteins such as p53 family members^26,27,34^, DNA-damage checkpoint proteins^30,31,35^, and key metabolic and stress proteins^28,29^ to mediate its tumor suppressor activities. While multiple mechanisms have been suggested to explain the tumor suppressive role of WWOX, there is currently no functional evidence for the exact role of WWOX in breast carcinogenesis.

We and others have previously reported that mammary gland epithelium (MGE)-specific *Wwox* deletion (*Wwox*^*ΔMGE*^) in B6/129 mixed genetic background does not result in a mammary tumor phenotype^36,37^. We therefore set out to determine whether somatic alteration in *Wwox* drives BLBC development in mammary tumor-susceptible mice: C3H/HeJ mice (shortly named C3H). We found that inactivation of *Wwox* in mammary gland epithelium in C3H mice results in mammary tumor formation resembling BLBC in human, as revealed by histological and molecular characterization of these tumors. This is the first mouse model that enables to directly study the role of WWOX in BLBC. Our data show that tumors of *Wwox* knockout or *Trp53* knockout are indistinguishable. At the molecular level, we show that WWOX loss results in reduced p53 activity either through destabilizing the genome, resulting in p53 loss and genomic instability, or through hindering p53 transcriptional function. We further demonstrate that deletion of *WWOX* and *TP53* co-occurs in breast cancer. Altogether, our results reveal WWOX, the gene product of FRA16D, as a *bona fide* breast cancer tumor suppressor with important functions in maintaining genome stability.

## Material and methods

### Mice

*Wwox*^*ΔMGE*^ mice (on B6/129 genetic background)^36^ were back-crossed onto the C3H/HeJ mice (shortly named C3H), a mammary tumor-susceptible genetic background, for seven rounds (~99% purity) generating *Wwox*^*ΔMMTV*^. Despite the lack of exogenous mouse mammary tumor virus (MMTV), virgin and breeding C3H female mice may still develop some mammary tumors later in life^38^. *Trp53*^*ΔMMTV*^ mice were generated by crossing *Trp53 loxP* mice^39^ with *MMTV-Cre* mouse (The Jackson Laboratory, #003553). Genotyping of *Wwox*, *Trp53* and Cre was performed using primers as detailed in Supplementary Table 3. All mice related experiments were approved by The Hebrew University Institutional Animal Care and Use Committee.

### Immunohistochemistry

Tissues were fixed in 4% formalin. Paraffin-embedded tissue sections were deparaffinized and rehydrated. Antigen retrieval was performed in 25 mM sodium citrate buffer PH 6.0 (for ER, PR, CK14, and gamma H2Ax) or EDTA buffer PH 8.0 (for WWOX) using pressurized chamber for 2.5 min. Endogenous peroxidase was blocked with 3% H_2_O_2_ for 15 min. The sections were then incubated with blocking solution (CAS Block) for 30 min to reduce non-specific binding followed by incubation with the primary antibody. Slides were subsequently incubated with horseradish peroxidase-conjugated anti-rabbit or anti-mouse immunoglobulin antibody for 30 min. The enzymatic reaction was detected in a freshly prepared 3,3 diamminobenzidine using DAB peroxidase kit (Vector laboratories) for several min at room temperature. The sections were then counterstained with hematoxylin. Eight tumors were stained for WWOX, ER, and CK14. Six tumors were stained for PR and γH2Ax.

### Isolation of primary mouse epithelial cells (MECs)

Mammary glands were isolated and minced from the indicated mice. For each gram tissue, 5 ml digestion mix [DMEM media, 10% fetal bovine serum, 1% pen-strep, 1:100 collagenase A (from stock 1.5 mg/ml) and 1:1,000 DNase-I (from 10 mg/ml stock)] was added and left for 1.5 h at 37 °C under moderate shaking (50 × g). MECs were separated from fat by differential centrifugation (three times) at 700×*g* for 30″. MECs were washed twice with PBS. In order to get rid of red blood cells, red blood cells lysis buffer was used.

### RNA extraction and RT-PCR

Total RNA was isolated using Tri-reagent. For RT-PCR, RNA (1 µg) was reversed transcribed using the QScript cDNA syntesis kit (Quantabio). Real-time PCR was done using SYBR Green PCR Master. Real-time PCR was performed using primers as indicated in Supplementary Table 4.

### Gene expression analysis

RNA from 4 *Wwox*^*ΔMMTV*^ mammary tumors, 2 *Trp53*^*ΔMMTV*^ mammary tumors, 4 double-knockout (*Wwox;Trp53*^*ΔMMTV*^) tumors 3 normal (prior to tumor formation) *Wwox*^*ΔMMTV*^ mammary epithelial cells (MECs) and 3 normal wild-type MECs was extracted and prepared for RNA sequencing. After poly-A cleanup, cDNA was synthesized. Libraries were made using KAPA Single-Indexed Adapter Kit (Illumina, Massachusetts, USA). Sequencing was performed using Next seq 500 (Illumina). Normalized gene expression values were log2-transformed and scaled by subtracting the gene expression means. Dendrograms were constructed using Euclidean distances and Complete linkage, and heat maps were generated with the “pheatmap” R package. Analysis was performed using all expressed genes: prior to the analysis, non-expressed genes (normalized expression value < 1 in all samples) were excluded, and expression levels were floored to 1. Data from double-knockout mice were processed with all other expression data, but their analysis is not included in the current study. For detailed bioinformatics analysis, see supplementary methods.

### E-karyotyping

E-karyotyping analysis was performed as previously described^40,41^. Briefly, normalized gene expression values were log2-transformed, non-expressed genes (log2 expression value < 1 in all samples) were excluded, and expression levels were floored to 1. The median expression value of each gene across normal samples was subtracted from the expression value of that gene in each normal and tumor sample, in order to obtain comparative values. Genes were ordered by their chromosomal location, and CNA profiles were then generated with the CGH-Explorer software (http://heim.ifi.uio.no/bioinf/Projects/CGHExplorer/), using the program’s piecewise constant fit (PCF) algorithm. The following set of parameters was used: Least allowed deviation = 0.25; Least allowed aberration size = 30; Winsorize at quantile = 0.001; Penalty = 12; Threshold = 0.01. Moving average plots were generated using the CGH-Explorer moving average fit tool.

### Comparison to other breast cancer mouse models

Gene expression data of five different GEMM types (Wnt, Myc, PyMT, Her2, and P53) from two studies^42,43^ (GSE23938 & GSE25488) were downloaded from the Gene Expression Omnibus (GEO) website (http://www.ncbi.nlm.nih.gov/geo). Gene expression values were log2-transformed, non-expressed genes (log2 expression value < 5.5 in > 20% of samples) were excluded, and expression levels were floored to 5. The median expression value of each gene across normal samples was subtracted from the expression value of that gene in each tumor sample, in order to obtain comparative values. Gene symbols were compared across the three datasets (the two previous studies and the current study), yielding a list of 5,897 genes present and expressed in all tumor samples. Batch effect was next removed using the COMBAT algorithm^44^. Unsupervised hierarchical clustering was performed on the batch-corrected expression values, using Euclidean distances and complete linkage. The Epithelial-Mesenchymal transition (EMT) gene set was downloaded from MSigDB^45^ (http://software.broadinstitute.org/gsea/msigdb). An EMT score was determined for each tumor sample as the sum of the comparative expression values of the expressed EMT genes. The average EMT score of each type of tumor was computed.

### Molecular subtype assignment

PAM50 centroid values were obtained from Parker et al.^46^, FPKM expression values of the PAM50 genes were log2-transformed, and the Spearman’s rank correlation between each sample and each subtype centroid was calculated. The class with the highest correlated centroid was assigned to each sample. In one p53 tumor, correlation values were similar for two subtypes (basal and lumB), and this tumor was therefore defined as mixed.

### Genomic DNA extraction and quantitative PCR

Tumors or normal MECs were minced using DNA lysis buffer (150 mM NaCl, 50 mM tris-HCL, 25 mM EDTA, 0.5% SDS and 5 µl proteinase K). After overnight incubation at 56 °C, phenol chloroform was added followed by centrifugation for 10 min. The upper phase was transferred to a new tube and followed by chloroform and isopropyl alcohol treatment. DNA was washed with 70% ethanol and eluted using TE buffer. Genomic DNA real-time PCR was performed using primers as indicated in Supplementary Table 5.

### Cell culture assays

ATCC breast cancer cell line MCF7 cells were cultured as in RPMI-1640 media supplied with 10% FCS, 1% pen-strep and 1% glutamine. The cells were mycoplasma free.

### CRISPR/CAS9 targeting WWOX expression

For knocking-out *WWOX* in MCF7 cells, lentiviruses were prepared using LentiCRISPR-V2 plasmid (Addgene plasmid # 52961) with sgRNAs (Supplementary Table 6) targeting WWOX exon 1(KO-1 cells) or exon 4 (KO-2 and KO-3 cells). From the infected cells pool, single clones were isolated. Parental cells and cells infected with empty vector were used as control.

### Colony formation assay

MCF7 cells were plated at a density of 500 cells in a 60-mm^2^ plate in triplicate. After 2 weeks the cells were fixed with 70% ethanol, stained with Giemsa and counted.

### Immunoblotting

Whole cell lysates were prepared using lysis buffer containing 50 mM Tris (pH 7.5), 150 mM NaCl, 10% glycerol, 0.5% Nonidet P-40, and protease inhibitors (1:100). Lysates were resolved on SDS/PAGE. Antibodies used were Rabbit polyclonal anti-GST-WWOX^11^ and mouse monoclonal anti-GAPDH and goat polyclonal anti-ATM, rabbit monoclonal anti-pATM, rabbit plyclonal anti-KAP, rabbit polyclonal anti-pKAP.

### WWOX overexpression in knockout cells

To prevent the CAS9 cut of the WWOX after the restoration, WWOX expressing lentivirus was mutated using QuikChange XL Site-Directed Mutagenesis Kit, Agilent Technologies, CA, USA. We mutated WWOX, at the guide RNA–identical sequence (CAS9-target sequence). The result sequence has different codons but codes for the same amino acids of the original sequence.

### Co-occurrence gene inactivation analysis

Gene-level mutation and copy number data of the METABRIC breast cancer dataset^47^ were downloaded from cBioPortal (www.cbiopotal.org). The number of tumors with a perturbation (homozygous deletion or mutation) in either *Trp53*, *Wwox* or both genes was determined. The statistical significance of co-occurrence was determined using a two-sided Fisher’s exact test.

### Survival analysis

Survival data of the METABRIC breast cancer dataset^47^ were downloaded from cBioPortal (www.cbiopotal.org). Tumors were separated into groups by their WWOX and p53 status: both genes WT, p53-perturbed (mutation, deletion or both), WWOX-perturbed (mutation, deletion or both), and both genes perturbed. Survival analysis was performed using the “survival” R package.

### Statistical analysis

Results were expressed as mean ± SD or ± SEM. The Student’s *t*-test was used to compare values of test and control samples. *P* < 0.05 indicated significant difference.

## Results

### Mammary-specific deletion of *Wwox* in *C3H* mice is associated with mammary tumorigenesis

Germline aged *Wwox*-heterozygous mice on C3H mammary tumor-susceptible genetic background develop mammary tumors with 50% penetrance^25^. To test whether somatic loss of WWOX in C3H mice could facilitate mammary tumorigenesis, *Wwox*^*ΔMGE*^ mice were back-crossed onto the C3H background for seven generations (N7/F1; ~99% purity) and incidence of mammary tumor formation was evaluated; these newly generated mice were named *Wwox*^*ΔMMTV*^.

**Fig. 1:**
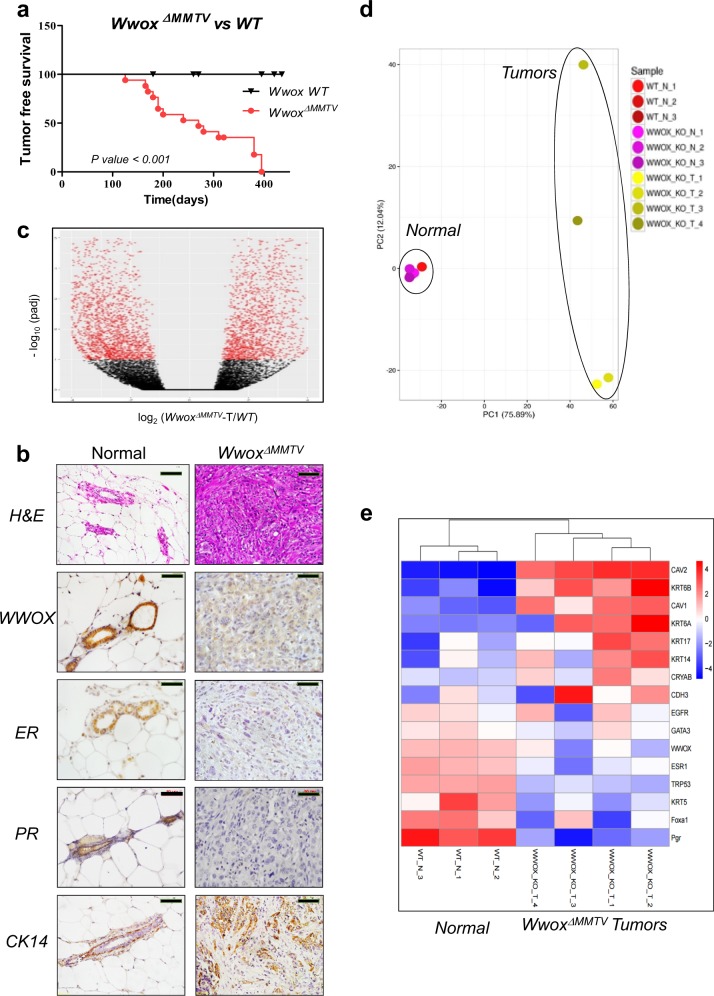
WWOX loss is associated with triple-negative breast cancer (TNBC) and particularly, the basal-like subtype. **a** Kaplan Meier analysis for *Wwox*^*ΔMMTV*^ mice as compared to wild-type mice. **b** Histological characterization of mammary tumors in *Wwox*^*ΔMMTV*^ and normal wild-type (WT) mice, using H&E staining and immunohistochemistry (anti-WWOX, anti-ER, anti-PR, and anti-CK14). Magnification bar represents 50 µm. **c** Volcano analysis for WT normal mammary tissue vs *Wwox*^*ΔMMTV*^ tumor tissues. **d** A principal component analysis (PCA) of normal mammary tissues from WT mice (*n* = 3), normal mammary tissues from *Wwox*^*ΔMMTV*^ mice (*n* = 3) and mammary tumors from *Wwox*^*ΔMMTV*^ mice (*n* = 4), based on their global gene expression patterns. **e** Top: unsupervised hierarchical clustering of three normal mammary tissues and four *Wwox*^*ΔMMTV*^ tumors, based on the expression of selected basal markers. Bottom: a corresponding heatmap, showing the expression levels of these genes

Monitoring of *Wwox*^*ΔMMTV*^ mice revealed that the majority (14/17, ~76%) developed mammary tumors with median latency of 270 days, while no mammary tumors were obtained in the control WT mice (Fig. 1a). Histological and pathological characterization of these *Wwox*^*ΔMMTV*^ tumors revealed that they are invasive ductal carcinoma, Grade III, with occasional lung metastases (2/14) (Fig. 1b, Supplementary Fig. S1a). Deletion of the *Wwox*^*fl/fl*^ alleles in these tumors was confirmed by immunohistochemistry using anti-WWOX antibody (Fig. 1b and Supplementary Fig. S1b). Immunohistochemical staining for ER and PR revealed that 100% of the tumors are ER/PR-negative, while ~60% stained positive for CK14 basal marker (Fig. 1b and Supplementary Fig. S1b). These findings are consistent with previously published data showing that WWOX loss in human is associated with TNBC and BLBC^9,12,14,25^. Altogether, these observations suggest that WWOX ablation on C3H mammary tumor-susceptible genetic background is associated with BLBC development. According to our knowledge, this is the first mouse model showing that somatic ablation of WWOX in adult mice is sufficient to drive mammary tumorigenesis.

### Molecular characterization of *Wwox*^*ΔMMTV*^ tumors

To molecularly characterize WWOX-deficient tumors, RNA from 4 *Wwox*^*ΔMMTV*^ mammary tumors, 3 normal (prior to tumor formation) *Wwox*^*ΔMMTV*^ mammary epithelial cells (MECs) and 3 normal wild-type MECs was extracted and analyzed using RNA sequencing. Differential gene expression analysis revealed that 5588 genes were differentially expressed in the mammary tumors compared to normal controls (Fig. 1c, Supplementary Table 1). Principal component analysis (PCA) of all samples revealed a clear separation of the tumors (*Wwox* _KO_T) from normal epithelial cells (Fig. 1d), whereas WT MECs (WT_N) and MECs isolated from *Wwox*^*ΔMMTV*^ pre-transformed tissues (*Wwox*_KO_N) clustered together (Fig. 1d). The differentially expressed genes were enriched for cancer-related pathways, including extracellular matrix, receptor interactions, focal adhesion and adherent junctions (Supplementary Table 2).

Consistent with our immunohistochemical analysis (Fig. 1b), RNA sequencing showed significant decrease in estrogen receptor (*Esr1*) and progesterone receptor (*Pgr*) transcripts, as well as upregulation of basal markers mRNA including *Ck14*, cytokeratin 6 (*Ck6*), cytokeratin 17 (*Ck17*), caveolin 1 (*Cav1*), caveolin 2 (*Cav2*), αB-Crystallin (*Cryab*) and P-cadherin (*Cdh3*)^9,25^ (Fig. 1e). Moreover, a significant reduction in RNA levels of *Foxa1*, known to repress the basal-like phenotype^48–50^, was observed (Fig. 1e), further confirming the BLBC nature of these tumors.

A major molecular subtype of TNBC is the claudin-low/mesenchymal-like subtype^51,52^, expressing low levels of tight junction proteins, including certain claudins and E-cadherin, and high levels of genes associated with epithelial-to-mesenchymal transition (EMT)^51^. Our RNA sequencing analysis showed an increase in expression levels of the majority of EMT markers but no decrease in expression levels of claudins in *Wwox*^*ΔMMTV*^ tumors compared to MECs (Supplementary Fig. S1c). Moreover, in a comparison of EMT scores between *Wwox*^*ΔMMTV*^ tumors and five other known mammary tumor mouse models, the *Wwox*^*ΔMMTV*^ model ranked among the highest, close to those of previously published Wnt and *Trp53* knockout models, known models to generate TNBC-like tumors (Supplementary Fig. S1d). Altogether, both immunohistochemistry and RNA sequencing data suggest that mammary tumors formed in *Wwox*^*ΔMMTV*^ mice resemble basal-like TNBC.

### *Wwox*^*ΔMMTV*^ tumors display hampered p53 expression and function

Similar to alterations of WWOX in BLBC, mutations in *TP53* were found in 88% of BLBC^52^. Moreover, conditional knockout of *Trp53* using *MMTV* and *WAP-Cre* in *C57BL/6* mice showed high percentage of tumor incidence, though at a later stage of life^53^. Using *MMTV-Cre* mice, high percentage (47%-100%) of *Trp53*^*f/f*^ mice developed mammary tumors with latency of 10–14.5 months^54^. These tumors were negative for both ER and PR and resulted in metastases in both liver and lung^54^. We therefore examined the expression of p53 and its target genes in *Wwox*^*ΔMMTV*^ tumors. Interestingly, our RNA sequencing analysis revealed a significant downregulation of *Trp53* levels (*P*-value = 0.0176) (Fig. 1e). To confirm this observation, an RT-PCR analysis was performed on additional mammary tumors obtained from *Wwox*^*ΔMMTV*^ mice, showing that *Trp53* levels were indeed downregulated in all tumors as compared to normal MECs or to archived tumors from *Wwox* wild-type or heterozygous mice^25^ (Supplementary Fig. S1e). Additionally, Global Gene Set Enrichment Analysis (GSEA) showed that the p53 pathway is suppressed in the *Wwox*^*ΔMMTV*^ tumors when compared to the normal samples (the genes that are upregulated when p53 is knocked-down are upregulated in the *Wwox*^*ΔMMTV*^ tumors, and the genes that are downregulated when p53 is knocked-down are downregulated in the tumors) (Supplementary Fig. S1f). Intriguingly, in one of three normal MECs isolated from *Wwox*^*ΔMMTV*^ mice, there was a significant decrease in *Trp53* gene expression (Supplementary Fig. S1g), suggesting that p53 downregulation might take place already at an early stage prior to mammary tumor development. To confirm impairment of p53 function, we measured levels of its target gene, *Cdkn1a*, and found a significant downregulation in the majority of *Wwox*^*ΔMMTV*^ tumors (Supplementary Fig. S1h) (*P*-value < 0.0001). These results imply that somatic loss of WWOX in mammary epithelium results in a reduced p53 activity to drive BLBC development.

### *Wwox*^*ΔMMTV*^ and *Trp53*^*ΔMMTV*^ tumors share similar patterns of gene expression and genomic instability

**Fig. 2:**
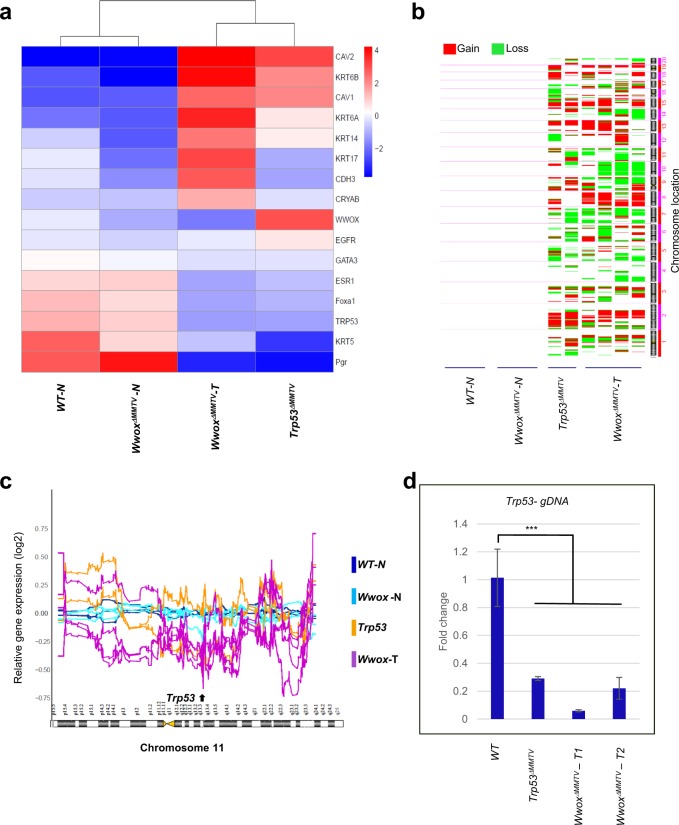
*Wwox*^*ΔMMTV*^ and *Trp53*^*ΔMMTV*^ tumors share common pattern of gene expression and genomic instability. **a** Top: unsupervised hierarchical clustering of normal mammary tissues from WT mice (*n* = 3), normal mammary tissues from *Wwox*^*ΔMMTV*^ mice (*n* = 3), mammary tumors from *Wwox*^*ΔMMTV*^ mice (*n* = 4) and mammary tumors from *Trp53*^*ΔMMTV*^ mice (*n* = 2), based on the expression of selected basal markers in each group. Bottom: a corresponding heatmap, showing the average expression levels of these genes in each group. Top: unsupervised hierarchical clustering of three normal mammary tissues and four *Wwox*^*ΔMMT****V***^ tumors, based on the expression of selected basal markers in each group. Bottom: a corresponding heatmap, showing the expression levels of these genes. **b** Expression-based karyotyping of the same samples shown in **a**. Copy number gains are shown in red; copy number losses are shown in green. **c** Gene expression moving average plots along *Trp53* locus in chromosome 11 of normal mammary tissues from WT mice (*n* = 3), normal mammary tissues from *Wwox*^*ΔMMTV*^ mice (*n* = 3), mammary tumors from *Wwox*^*ΔMMTV*^ mice (*n* = 4) and mammary tumors from *Trp53*^*ΔMMTV*^ mice (*n* = 2). These expression patterns suggest that three out of four *Wwox*^*ΔMMTV*^ mice have lost a copy of chromosome 11, which includes the *Trp53* gene. **d** Quantitative PCR performed on genomic DNA of WT tissue, one *Trp53*^*ΔMMTV*^ tumor and two *Wwox*^*ΔMMTV*^ tumors, for normalization primers specific for *Trim2* (didn’t not affect by *Wwox* loss) were used. Error bars represent standard deviation

The previous observations prompted us to determine whether inactivation of *Wwox* resembles the effect of *Trp53* inactivation in mammary gland epithelium. We therefore generated *Trp53* mammary conditional knockout mice on B6/129 mixed background (named *Trp53*^*ΔMMTV*^) and examined mammary tumor formation. While *Wwox*^*ΔMGE*^ mice did not develop tumors, we found that *Trp53*^*ΔMMTV*^ mice developed mammary tumors with latency of 262.5 days (*P*-value = 0.007) and 45% penetrance. Histological and pathological characterization of *Trp53*^*ΔMMTV*^ mammary tumors revealed an invasive ductal carcinoma - Grade III that displays ER/PR negative expression and CK14 positive expression (Supplementary Fig. S2a). These results suggest that deregulation of WWOX and p53 share similar functions in driving BLBC mammary tumor development. Indeed, when comparing *Trp53*^*ΔMMTV*^ tumors to *Wwox*^*ΔMMTV*^ tumors, the two tumor groups shared similar upregulation of the basal markers (Supplementary Fig. S2a and Fig. 2a) and enhanced expression of EMT markers (Fig. S2b). Interestingly, *Trp53*^*ΔMMTV*^ tumors showed no or very low levels of WWOX protein as assessed by immunohistochemistry (Supplementary Fig. S2a). In contrast, *Wwox* RNA levels in these tumors were markedly upregulated as revealed by RNA sequencing and qRT-PCR analyses (Fig. 2a and data not shown), possibly suggesting a compensatory effect. These data suggest that mutual inactivation of both p53 and WWOX takes place during mammary tumor formation.

To further characterize these tumors, we used their gene expression profiles to classify them according to the PAM50 breast cancer intrinsic subtypes^46,55^. Four out of four WWOX-KO tumors and one out of two p53-KO tumors best matched the basal subtype (Supplementary Table 7). The other p53-KO tumor displayed a mix of basal-like and LumB expression signatures, consistent with previously published analyses of p53 mouse models^46,55^. These findings further confirm that WWOX loss is associated with BLBC formation.

We further examined whether the global gene expression profile of our *Wwox*^*ΔMMTV*^ model is similar to those of previously published mammary-specific p53 knockout models. Indeed, the *Wwox*^*ΔMMTV*^ model clustered together with the *Trp53* KO models from a previous study (GSE23938)^42^ (Supplementary Fig. S2c).

To better characterize our mammary tumor models, we performed expression based-karyotyping analysis (e-karyotyping)^40,41^ to explore the chromosomal landscapes of the tumors (Fig. 2b). Expression-based analysis of copy-number changes in both *Wwox*^*ΔMMTV*^ *Trp53*^*ΔMMTV*^ models showed that all tumors exhibit high prevalence of copy-number alterations (Fig. 2b). Consistent with chromosomal aberrations and genomic instability, we observed high levels of γH2AX staining, a surrogate marker of DNA double strand breaks and repair signaling, in tumors of both groups (Supplementary Fig. S2d). Altogether, *Wwox*^*ΔMMTV*^ and *Trp53*^*ΔMMTV*^ mice developed mammary tumors with very similar histologies, gene expression profiles, and genome instability patterns.

### *Wwox*^*ΔMMTV*^ tumors are p53 deficient

The similar molecular features of the two models and the reduction of *Trp*53 mRNA levels in *Wwox*^*ΔMMTV*^ tumors suggest that changes in p53 levels may occur due to DNA gene deletion or due to transcriptional attenuation. While all *Wwox*^*ΔMMTV*^ tumors have significantly decreased *Trp53* gene expression, our expression-based copy-number analysis suggested that three of the four *Wwox*^*ΔMMTV*^ tumors analyzed presented a large-scale copy-number loss in a region that includes the *Trp53* gene, which may explain its reduced expression and the overall similarity between the two models (Fig. 2c). We therefore directly addressed this question, by performing quantitative real-time PCR on genomic DNA extracted from the *Wwox*^*ΔMMTV*^ and the *Trp53*^*ΔMMTV*^ tumors. DNA isolated from wild-type and normal *Wwox*^*ΔMMTV*^ MECs was used as a control. As expected, *Trp53*^*ΔMMTV*^ samples had a deletion in exons 2–10 of the *Trp53* gene, which were originally targeted by the loxP sites. In line with the e-karyotype analysis, we also noticed a significant decrease in *Trp53* genomic DNA levels in *Wwox*^*ΔMMTV*^ tumors, indicating that the reduction of *Trp53* expression was indeed due to its genomic loss (Fig. 2d) *P*-value < 0.005). We also examined the DNA status of genes located upstream and downstream of *Trp53* region, and found reduced genomic levels of *Wrap53* (*P*-value < 0.001) (upstream), as well as of *Atp1b2* (*P*-value < 0.001 for T2 and T3)*, Shbg* (*P*-value < 0.001)*, Sat2* (*P*-value < 0.001) and *Fxr2* (*P*-value < 0.001) genes (downstream) in the *Wwox*^*ΔMMTV*^ tumors, further suggesting that loss of *Wwox* resulted in genetic loss of the *p53* locus (Supplementary Fig. S2e–g). Consistent with this later observation, p53 expression level in *Wwox*^*ΔMMTV*^ model was as low as in the *Trp53*^*ΔMMTV*^ model (Fig. 2a).

### WWOX knockout in MCF7 cells using CRISPR

**Fig. 3:**
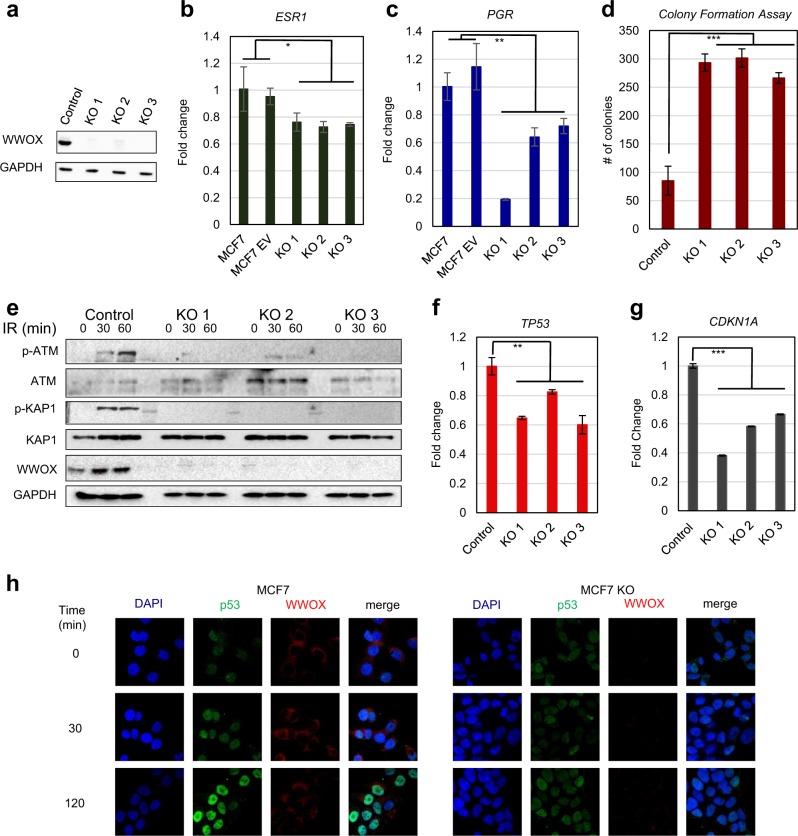
*WWOX* knockout, using CRISPR system, in the human MCF7 breast cancer cell line. **a** Western blot validates *WWOX* knockout (KO) in MCF7 cells compared to control cells; three clones are shown. **b**, **c** Quantitative RT-PCR for estrogen receptor gene (*ESR1*) (**b**) and for progesterone receptor gene (*PGR*) in the three *WWOX* KO clones in **a**. For both **b** and **c**, MCF7 parental cells and cells infected with empty vector were used as a control. **d** Colony formation assay in the three KO clones. **e** Immunoblot analysis for DNA-damage checkpoint proteins after gamma irradiation (10 Gy). **f** qPCR for *TP53* and its targets *CDKN1A* (p21) in the WWOX-KO clones. Error bars represent standard deviation. *P-value < 0.05, **<0.01, ***<0.001. **h** Immunofluorescence for WWOX and p53 before and after gamma irradiation (10 Gy). Magnification bar represents 20 µm

To further determine the impact of *WWOX* deletion, we used CRISPR technology to knockout WWOX in a WWOX-positive breast cancer cell line. Since TNBC cell lines express very low levels of WWOX^56^, we knocked-out WWOX in MCF7, an ER+ cell line that expresses high levels of WWOX and harbors wild-type p53^56,57^. Complete WWOX knockout was confirmed in several clones of WWOX-KO MCF7 cells by immunoblot analysis (Fig. 3a). Consistent with previously published data on WWOX knockdown and reduced hormone receptor levels^25^, WWOX-KO MCF7 cells displayed reduced ER and PR transcript levels (Fig. 3b and c) (*P*-value < 0.05 and < 0.01 respectively). These cells also exhibited a significantly increased survival capability, as assessed by a colony formation assay (Fig. 3d).

WWOX has been shown to play a direct role in the DNA damage response (DDR)^31^. To determine the consequence of WWOX deletion on DDR signaling, we tested checkpoint protein activation and p53 status in MCF7-KO cells upon ionizing radiation (IR). WWOX knockout clones showed reduced or no phosphorylation of both ATM and its target protein KAP1 upon DNA damage, suggesting an impaired DDR in these clones (Fig. 3e). Moreover, WWOX-deficient MCF7 cells exhibited reduced induction of nuclear p53 and decreased expression of p53 target genes, p21 (Fig. 3f–h) and PUMA (Supplementary Fig. S3a). However this reduction was not due to genomic loss. Importantly, WWOX over-expression, using CRISPR-untargetable WWOX mutant, rescued these phenotypes (Supplementary Fig. S3b-d), confirming that the observed changes were due to on-target perturbation of WWOX. Altogether, WWOX loss is associated with impaired p53 function, enhanced survival and impaired DDR in MCF7 cells.

### Co-occurrence of *WWOX* and *TP53* deletion in breast cancer

**Fig. 4:**
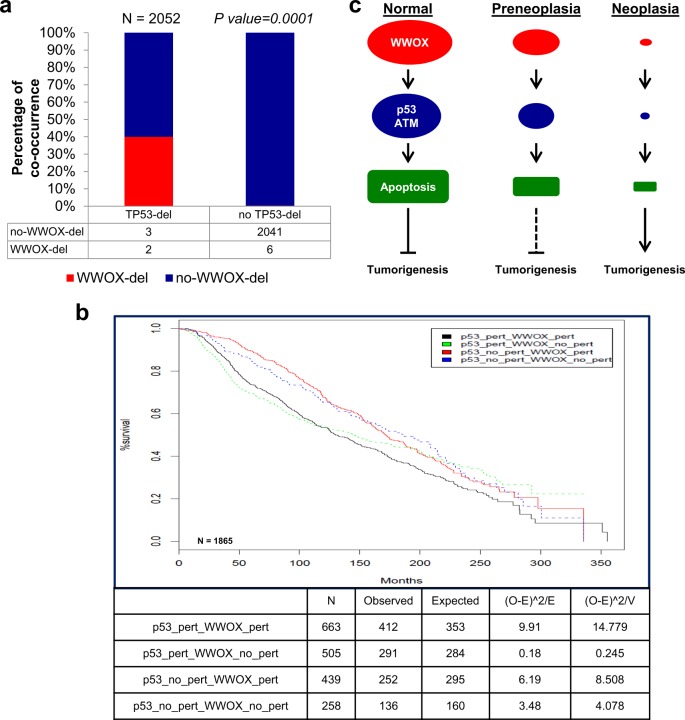
Co-occurrence and combined perturbation in WWOX and p53 predicts worse survival in breast cancer patients. **a** Bar plots present the prevalence of *WWOX* inactivation (homozygous deletion) in human tumors (METABRIC dataset) that have lost *TP53* (homozygous deletion) compared to tumors that have not. *P*-value = 0.0001, two-sided Fisher’s exact test. **b** Kaplan–Meier plots of the survival of breast cancer patients from the METABRIC dataset, based on their TP53-WWOX status: TP53-perturbed/WWOX-WT (green), TP53-WWOX-perturbed (black), TP53-WT/WWOX-WT (blue), TP53-WT/WWOX-perturbed (red). *P*-value = 2*E−4, Chi-square test. Note that while the survival curves are mostly affected by the status of TP53, poorer survival is observed in patients with perturbation of both genes. **c** WWOX loss-induced p53 loss model for TNBC development. In normal cells undergoing stress, WWOX cooperates with p53, and other DDR proteins such as ATM, leading to efficient DDR (apoptosis or DNA repair). Deletions or other alteration in *WWOX* alleles occurs in early preneoplastic lesions, as in DCIS, and leads to impaired DDR resulting in destabilization of the genome, hence leading to compromised function of key tumor suppressor genes, such as p53 and ATM. This would result in further genomic instability, enabling cells to overcome the tumor barrier and embark on cancer evolution.

*WWOX* and *TP53* are both commonly perturbed in breast cancer. To determine the human relevance of combined alteration of WWOX and p53, we analyzed their mutual perturbation in the METABRIC dataset (www.cbioportal.org;^47,58^ 2509 patients samples). Surprisingly, we found that genetic alterations of *WWOX* were a rare event in this dataset of human patients, despite multiple evidence for WWOX protein expression reduction in clinical samples of breast cancer, and in TNBC in particular^12,13^. Despite their rareness, however, we found a very significant co-occurrence of *WWOX* homozygous deletion and *TP53* homozygous deletion (*p* = 0.0001 in a two-tailed Fisher’s exact test; Fig. 4a). Furthermore, while *TP53* perturbation was associated with poorer survival as expected (Fig. 4b), genetic perturbation of *WWOX* was not associated with poorer survival of TP53-perturbed patients (Fig. 4b). These data therefore support the idea that p53 and WWOX cooperate in human breast cancer tumorigenesis, and that loss of WWOX promotes tumorigenesis through the perturbation of *TP53*.

## Discussion

BLCB is a heterogeneous, highly aggressive and difficult to treat tumor type^59–62^. We found that *WWOX* loss is associated with worse prognosis in BLBC, and that its targeted deletion in murine mammary epithelium leads to mammary tumors resembling BLBC. In a previous study, we noted that WWOX haploinsufficiency in C3H background promotes BLBC^25^. These observations are confirmed in the current study using a new mouse model harboring floxed-*Wwox* alleles. While none of control WT mice on the C3H background developed mammary tumors, somatic loss of *Wwox* alleles in this genetic background promoted BLBC-like tumors (Fig. 1). Although developed in C3H background, these tumors clustered together with tumors from *Trp53*^*ΔMMTV*^ mice (in B6/129 background), suggesting that these tumors are very similar and that a functional crosstalk between WWOX and p53 is critical to antagonize BLBC. Our findings suggest that WWOX deletion by itself might not be enough for BLBC development, and that a second hit facilitated in the C3H background, like perturbations in p53 signaling or locus, is required. Indeed, WWOX inactivation was associated with *Trp53* gene instability and/or impaired DNA-damage checkpoint protein activation. Deletion of *Wwox* or *Trp53* resulted in aggressive BLBC-like tumors. RNA profiling of mammary tumors of the different mouse models revealed similar patterns, though not identical, of differential gene expression, highlighting upregulation of basal-cell markers and EMT genes. Furthermore, co-occurrence of mutations in the *TP53* and *WWOX* tumor suppressor genes is commonly seen in patients. Overall, WWOX functions as a mammary tumor suppressor, likely through mediating genome stability in a p53-dependent mechanism, and its loss of function in mammary epithelium reproduces several important features of BLBCs.

TNBC and BLBC tumors commonly harbor deleted or mutated p53, and mouse modeling of *Trp53* dysregulation is associated with BLBC tumor formation^53,55,60^. The *WWOX* gene has been originally cloned due to its common inactivation in breast cancer^5,6^. Several subsequent studies have shown that WWOX expression is reduced or absent in breast cancer, particularly TNBC and BLBC^9,13,14^, and that its overexpression in breast cancer cells suppresses tumor growth^17^ and enhances apoptosis mediated by activation of the p53 family proteins^26,27,34^. These results suggested that WWOX could act as a tumor suppressor, though its localization within FRA16D questioned this function.

Common fragile sites (CFSs) are regions that appear in vitro as gaps or breaks in metaphase chromosomes of cells exposed to partial inhibition of DNA replication^63^. The significance of CFSs was recently highlighted, as many regions associated with these sites display homozygous and hemizygous deletions in human cancer^64,65^ hence accusing them to be the “weakest link” of the genome and to be responsible for genomic instability^63,66,67^. Furthermore, it was suggested that some of these regions might act as early warning sensors for DNA damage since chromosomal aberrations within CFSs were detected in experimentally-induced skin hyperplasia and in early stages of lung cancer^68,69^. By contrast, emerging evidence has shown that gene products of CFSs, at least some, may play direct roles in the DNA damage response (DDR), hence questioning their exact role in the carcinogenesis process^70–72^. For example, the fragile histidine triad (*FHIT*) gene was recurrently found lost in a plethora of human tumors, including TNBC^13^, and its deregulated expression was associated with unstable genome (reviewed in the ref. ^71^). In addition, recent studies revealed that WWOX levels are induced upon DNA damage and that WWOX interacts with DNA damage checkpoint proteins ATM^31^ and BRCA1^35^ to regulate DNA repair in breast cancer cells. These results argue against a passenger role of WWOX in breast cancer. These recent observations defining WWOX as a direct player in the DDR^30,31,35^ led us to pursue our hypothesis and test whether specific targeted deletion of *Wwox* in murine mammary epithelium results in mammary tumor formation. Our results provide clear evidence that WWOX loss results in spontaneous deregulation of the *Trp53* gene locus, leading to impaired DNA damage response and mammary tumors resembling human BLBC. This is the first *in vivo* evidence linking somatic WWOX deregulation in a mammary cell with spontaneous mammary tumorigenesis.

We show that WWOX inactivation results in destabilization of the genome and p53 loss, although the direct mechanisms of this remains to be determined. It is possible that the p53 genomic region is not directly targeted, but that WWOX loss leads to mild genomic instability, and the cells that lose the *Trp53* locus are strongly selected for. Should this be true, we would expect cells harboring both WWOX and p53 alterations to have a greater advantage in cell growth and transformation and hence greater and accelerated tumor formation. Our findings might suggest that both WWOX and p53 function in the same pathway, and hence their deregulation in cancer is expected to be mutually exclusive. Surprisingly, we found that co-occurrence of altered *WWOX* and *TP53* is common in breast cancer, suggesting that WWOX may have additional functions other than the DDR. In line with this cooperation, combined WWOX and p53 loss has been also shown to be associated with aggressive osteosarcoma formation^73^.

Our findings in vitro also support an important role of WWOX on p53 function. Modeling WWOX knockout in MCF7 cells didn’t affect the genomic locus of *TP53* but rather had a significant transcriptional repression and reduced activity of p53 (Fig. 3f–h). Therefore, WWOX may also act as a positive regulator of p53 transcription, potentially through its ability to bind transcription factors via its WW domains^74^.

Our findings prompt us to propose a model (Fig. 4c) by which WWOX cooperates with p53 under stress conditions, leading to enhanced apoptosis as previously reported^26,75^, and this cooperation is part of the tumorigenesis barrier. During early stages of breast cancer development, WWOX is reduced or lost, as documented in hyperplasia and DCIS lesions^11^, either genetically or as a result of epigenetic silencing. WWOX deregulation leads to impaired DDR, and results in destabilization of the genome, thus leading to compromised function of key tumor suppressor genes, such as p53. This would result in further genomic instability, enabling cells to overcome the tumor barrier and embark on cancer evolution. WWOX could also modulate p53 and other important signaling molecules by affecting their transcription or transactivation function and hence impact the carcinogenesis process. Our model further supports a scenario where CFSs and their gene products might have far reaching roles in driving human malignancies.

### GEO data availability

The RNA sequencing data in this publication have been deposited in NCBI's Gene Expression Omnibus and are accessible through GEO Series accession number GSE117387.

## Electronic supplementary material

Supplementary Figures

Supplementary Figure Legends

Supplemental Table 1

Supplemental Table 2-7
