# Supplementary material for: Somatic loss of WWOX is associated with TP53 perturbation in basal-like breast cancer
Source: Cell Death Dis. 2018 Aug 6;9(8):832. doi: 10.1038/s41419-018-0896-z (PMC6079009; doi:10.1038/s41419-018-0896-z)
Supplement: Supplementary file 1 — Supplementary Figures [file 41419_2018_896_MOESM1_ESM.pptx]

## Slide 1
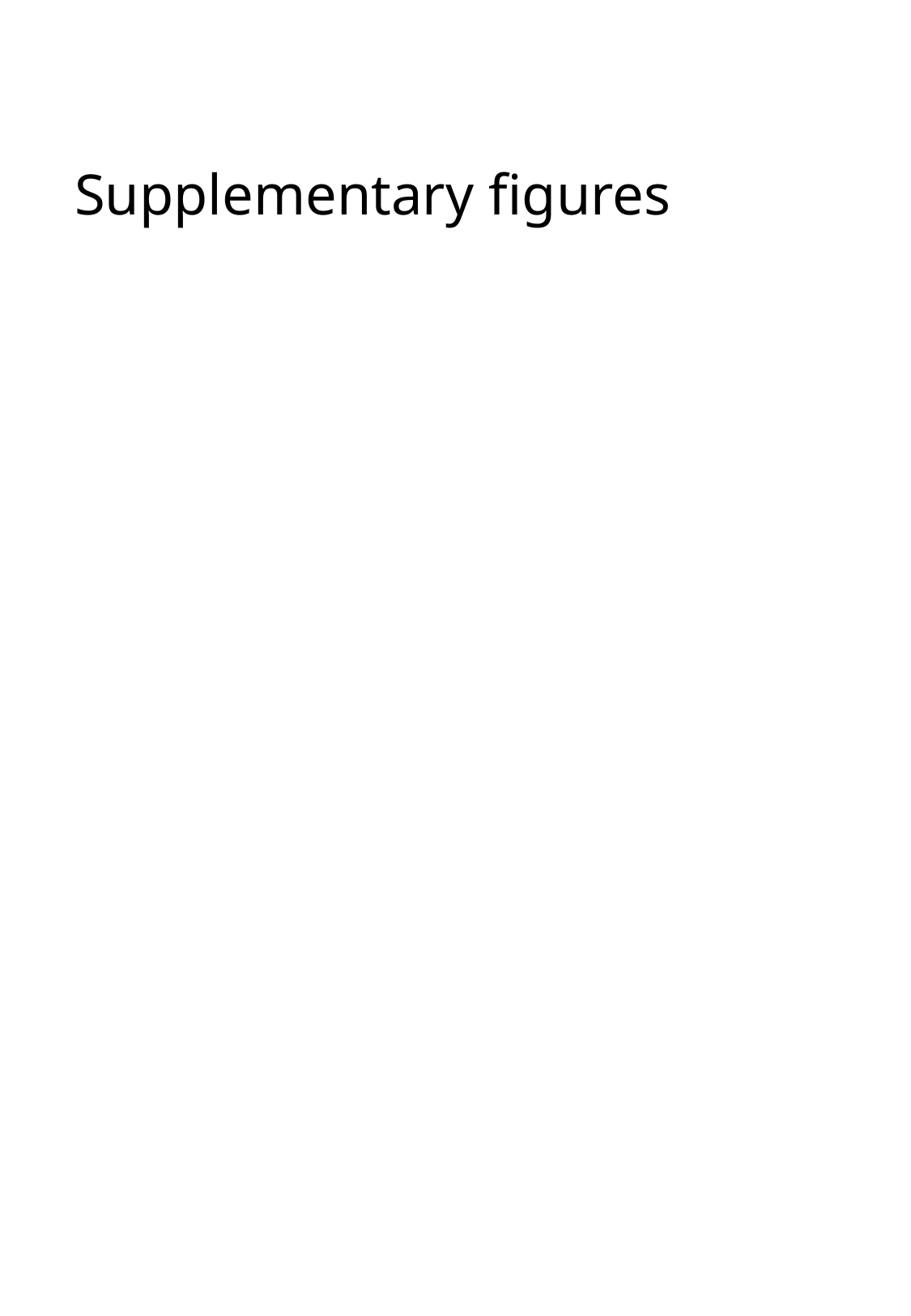

# Supplementary figures

## Slide 2
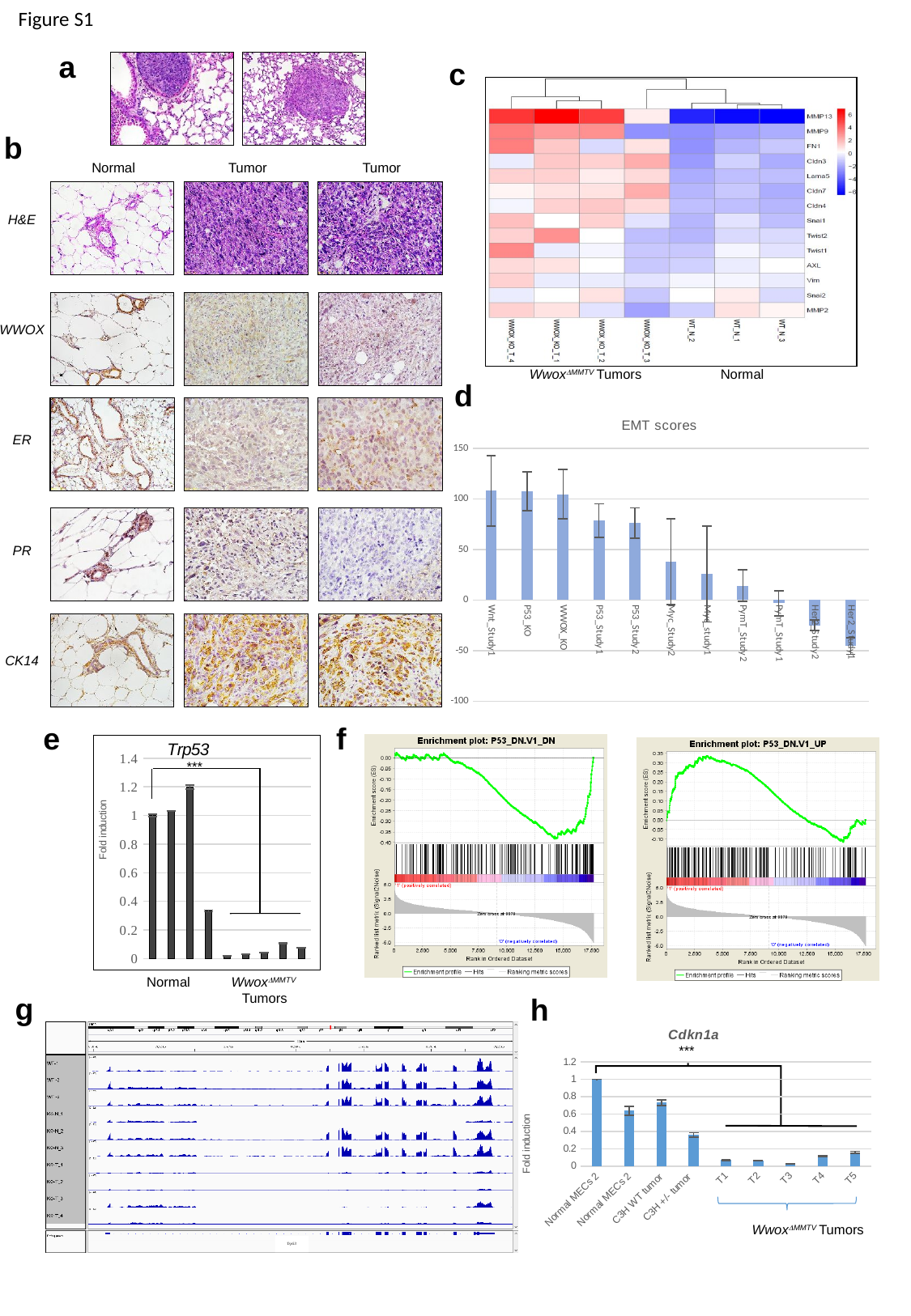

Figure S1
a
c
Normal
WwoxΔMMTV Tumors
b
Normal
Tumor
Tumor
H&E
WWOX
ER
PR
CK14
d
### Chart: EMT scores
| Category | |
|---|---|
| Wnt_Study1 | 107.98411305675 |
| P53_KO | 107.5306839255 |
| WWOX_KO | 104.71003478725 |
| P53_Study1 | 78.4731199647143 |
| P53_Study2 | 76.19461967057141 |
| Myc_Study2 | 37.92469805024999 |
| Myc_study1 | 25.7380186265 |
| PymT_Study2 | 14.23389201775 |
| PymT_Study1 | -3.1683102355 |
| Her2_Study2 | -25.0850450646 |
| Her2_Study1 | -45.32950364960001 |e
f
### Chart: Trp53
| Category | |
|---|---|
| WT mixed | 1.000019378365441 |
| Normal 1 | 1.033592560343906 |
| C3H WT DMBA | 1.196704579119411 |
| M22 | 0.334280531097227 |
| C3H KO 1 | 0.0191634927402282 |
| C3H KO 2 | 0.0318062958752418 |
| C3H KO 3 | 0.0413110758606799 |
| C3H KO 4 | 0.108862609925121 |
| C3H KO 5 | 0.0762521696677597 |
***
Normal
WwoxΔMMTV
Tumors
g
h
### Chart: Cdkn1a
| Category | |
|---|---|
| Normal MECs 2 | 1.000000160151011 |
| Normal MECs 2 | 0.637468247558473 |
| C3H WT tumor | 0.731027020578504 |
| C3H +/- tumor
 | 0.359197059674243 |
| T1 | 0.0689097037750127 |
| T2 | 0.0640330119665071 |
| T3 | 0.0242779137648468 |
| T4 | 0.11332905139918 |
| T5 | 0.15828632464558 |***
WwoxΔMMTV Tumors
Trp53

## Slide 3
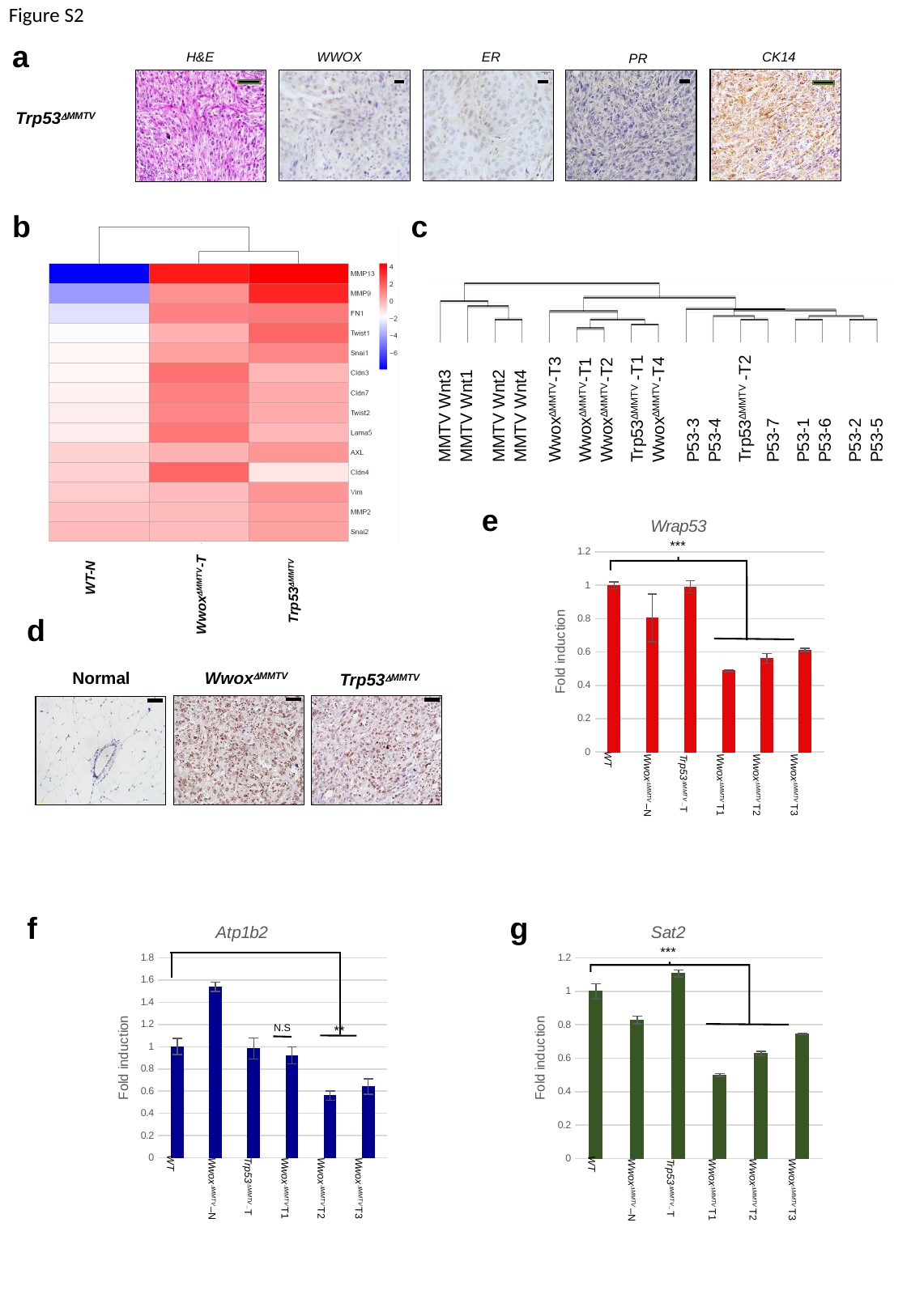

Figure S2
a
H&E
WWOX
ER
CK14
PR
Trp53ΔMMTV
c
b
Trp53ΔMMTV
WwoxΔMMTV-T
WT-N
MMTV Wnt3
MMTV Wnt1
MMTV Wnt2
MMTV Wnt4
Wwox∆MMTV-T3
Wwox∆MMTV-T1
Wwox∆MMTV-T2
Trp53∆MMTV -T1
Wwox∆MMTV-T4
P53-3
P53-4
Trp53∆MMTV -T2
P53-7
P53-1
P53-6
P53-2
P53-5
e
### Chart: Wrap53
| Category | |
|---|---|
| WT | 1.000139917730187 |
| WWOX KO | 0.804542084157524 |
| P53 KO tumor | 0.990339307361665 |
| WWOX KO T1 | 0.487922417500242 |
| WWOX KO T2 | 0.560686640026179 |
| WWOX KO T3 | 0.612076407514301 |***
WT
Trp53ΔMMTV _T
WwoxΔMMTV T1
WwoxΔMMTV T2
WwoxΔMMTV T3
WwoxΔMMTV –N
d
Normal
WwoxΔMMTV
Trp53ΔMMTV
f
g
### Chart: Atp1b2
| Category | |
|---|---|
| C3H WT | 1.001768425475317 |
| C3H KO Normal | 1.539027949372948 |
| P53 KO tumor | 0.982783278650547 |
| WWOX KO T1 | 0.921447651087491 |
| WWOX KO T2 | 0.561396189607789 |
| WWOX KO T3 | 0.642217660419028 |N.S
**
WT
Trp53ΔMMTV _T
WwoxΔMMTV T1
WwoxΔMMTV T2
WwoxΔMMTV T3
WwoxΔMMTV –N
### Chart: Sat2
| Category | |
|---|---|
| WT | 1.000688459895038 |
| WWOX KO | 0.82877651164225 |
| P53 KO tumor | 1.108163665707602 |
| WWOX KO T1 | 0.499932747424351 |
| WWOX KO T2 | 0.628162663984714 |
| WWOX KO T3 | 0.744670274266461 |***
WT
Trp53ΔMMTV _T
WwoxΔMMTV T1
WwoxΔMMTV T2
WwoxΔMMTV T3
WwoxΔMMTV –N

## Slide 4
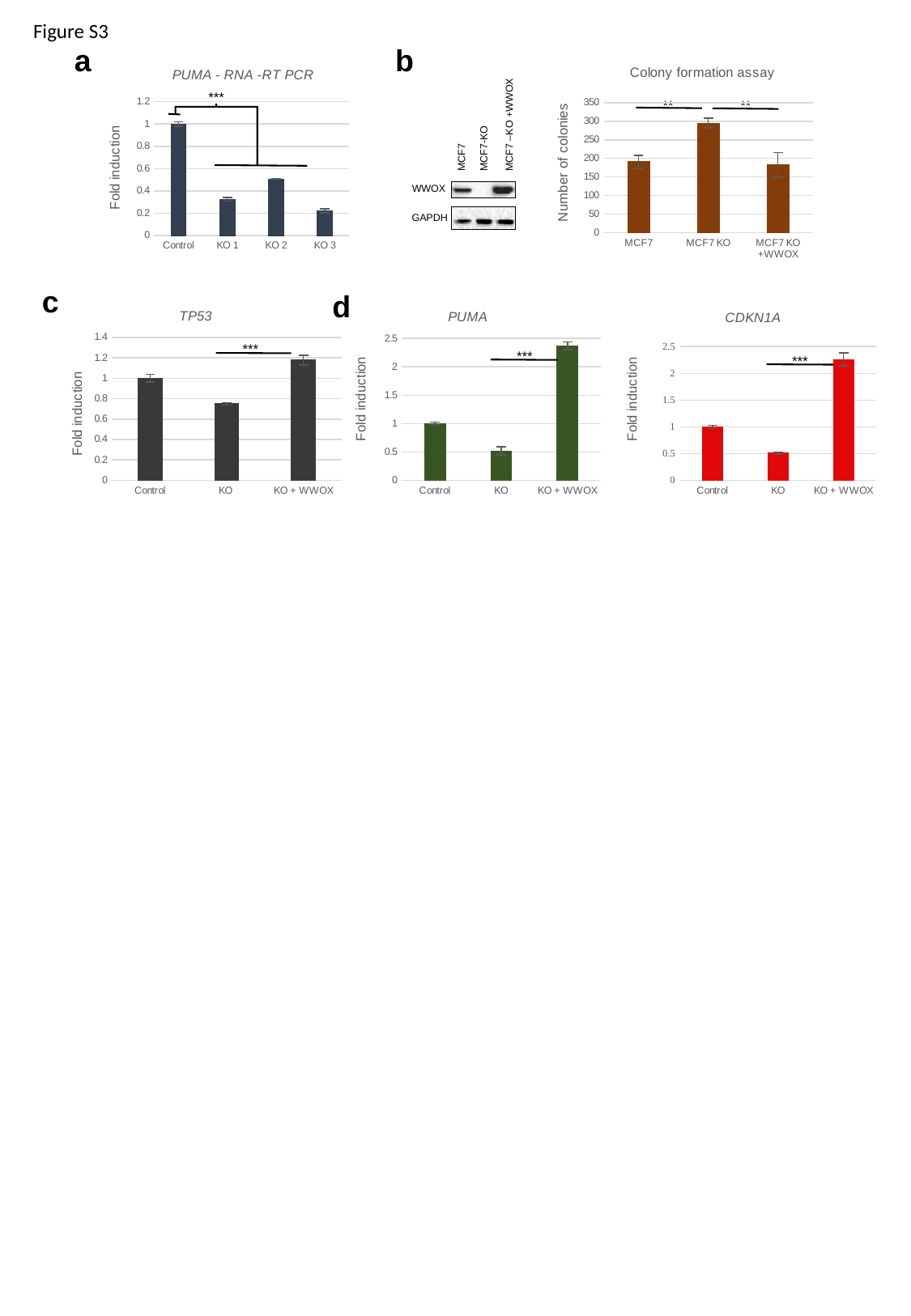

Figure S3
a
### Chart: PUMA - RNA -RT PCR
| Category | |
|---|---|
| Control | 1.000139557582129 |
| KO 1 | 0.324464460930075 |
| KO 2 | 0.505345680229861 |
| KO 3 | 0.21961933766909 |b
MCF7-KO
MCF7 –KO +WWOX
MCF7
WWOX
GAPDH
### Chart: Colony formation assay
| Category | |
|---|---|
| MCF7 | 190.6666666666666 |
| MCF7 KO | 295.0 |
| MCF7 KO +WWOX | 182.6666666666666 |**
**
***
c
### Chart: TP53
| Category | |
|---|---|
| Control | 1.000498162135956 |
| KO | 0.748712834846787 |
| KO + WWOX | 1.177434612262816 |***
d
### Chart: PUMA
| Category | |
|---|---|
| Control | 1.000234260160271 |
| KO | 0.516864284491915 |
| KO + WWOX | 2.366715453440531 |***
### Chart: CDKN1A
| Category | |
|---|---|
| Control | 1.00030080017379 |
| KO | 0.511871853416426 |
| KO + WWOX | 2.260428848588834 |***
