# Supplementary material for: Somatic loss of WWOX is associated with TP53 perturbation in basal-like breast cancer
Source: Cell Death Dis. 2018 Aug 6;9(8):832. doi: 10.1038/s41419-018-0896-z (PMC6079009; doi:10.1038/s41419-018-0896-z)
Supplement: Supplementary file 2 — Supplementary Figure Legends [file 41419_2018_896_MOESM2_ESM.docx]

**Supplemental Figure Legends**

**Fig. S1: Molecular characterization of WwoxΔMMTV tumors, related to Fig. 1 and 2.**

a. H&E staining for lung metastasis in *WwoxΔMMTV* mice with mammary tumors. b. Histological characterization of mammary tumors in *WwoxΔMMTV* and normal wild-type (WT) mice, using H&E staining and immunohistochemistry (anti-WWOX, anti-ER, anti-PR and anti-CK14). c.Top: unsupervised hierarchical clustering of three normal mammary tissues and four *WwoxΔMMTV* tumors, based on the expression of selected EMT markers. Bottom: a corresponding heatmap, showing the expression levels of these genes. d. The two models generated in the current study (*WwoxΔMMTV* and *Trp53ΔMMTV*) and nine additional models representing five distinct GEMM types (Wnt, Myc, PyMT, Her2 and P53) are ranked by their EMT scores. Study 1=GSE23938, study 2=GSE25488. Error bars represent standard error of the mean. e. Quantitative RT- PCR for the Trp53 gene in *WwoxΔMMTV* tumors compared to control samples. Error bars represent standard deviation. P-value <0.001. f. GSEA analysis (using both the "Hallmark" and the "Oncogenic" MsigDB gene sets) for *WwoxΔMMTV* in comparison to normal cells. g. IGV software image for the Trp53 gene in the analyzed Wildtype, *WwoxΔMMTV* normal and tumor tissue. h. Quantitative RT-PCR for the p53 target Cdkn1a compared to normal MECs or to archived tumors from p53 wildtype or heterozygous mice. Error bars represent standard deviation. P-value < 0.001.

**Fig. S2: Wwox loss is associated with Trp53 perturbation, related to Figures 1 and 2.**

a. Histological characterization of mammary tumors in Trp53ΔMMTV mice, using H&E staining and immunohistochemistry (anti-WWOX, anti-ER, anti-PR and anti-CK14). Magnification bar represents 20 and 50 µm. b. Top: unsupervised hierarchical clustering of normal mammary tissues from WT mice (n=3), normal mammary tissues from *WwoxΔMMTV* mice (n=3), mammary tumors from *WwoxΔMMTV* mice (n=4) and mammary tumors from *Trp53ΔMMTV* mice (n=2), based on the expression of selected basal markers in each group. Bottom: a corresponding heatmap, showing the average expression levels of these genes in each group. c. An unsupervised hierarchical clustering of mammary tumors, representing the two models generated in the current study (*Trp53ΔMMTV* and *WwoxΔMMTV*) and previously published Trp53-KO models (GSE23938). The Wnt data are taken from the same study. d. γH2Ax staining. Scale bar represents 50 micrometer. e, f and g. DNA-quantitative PCR for genes *upstream* (*Wrap53*) and downstream (*Atp1b2* and *Sat2*) of *Trp53* locus. Three *WwoxΔMMTV* tumors were checked and compared to normal mammary tissue of wildtype, normal mammary tissue of *WwoxΔMMTV* and *Trp53ΔMMTV* mammary tumor. P-value * <0.05, ** < 0.01, *** <0.001.

**Fig. S3: Effect of WWOX overexpression (Using CRISPR untargetable WWOX mutant) in WWOX-KO MCF7 cells, related to Fig. 3.**

a. qPCR for the p53 target PUMA in MCF7 cells. Error bars represent standard deviation. P value < 0.001. Effect of WWOX overexpression on survival (b), TP53 (c) and its targets expression (d). Error bars represent standard deviation. P value < 0.001.
