# Supplementary material for: Somatic loss of WWOX is associated with TP53 perturbation in basal-like breast cancer
Source: Cell Death Dis. 2018 Aug 6;9(8):832. doi: 10.1038/s41419-018-0896-z (PMC6079009; doi:10.1038/s41419-018-0896-z)
Supplement: Supplementary file 4 — Supplemental Table 2-7 [file 41419_2018_896_MOESM4_ESM.docx]

**Supplementary Table 2: Up-regulated pathways in *Wwox^ΔMMTV^* tumors compared to WT normal tissue.**

| [**Term**](https://david.ncifcrf.gov/chartReport.jsp?d-16544-s=2&d-16544-o=2&d-16544-p=1&annot=45) | [**Count**](https://david.ncifcrf.gov/chartReport.jsp?d-16544-s=5&d-16544-o=1&d-16544-p=1&annot=45) | [**P-Value**](https://david.ncifcrf.gov/chartReport.jsp?d-16544-s=7&d-16544-o=1&d-16544-p=1&annot=45) |
| --- | --- | --- |
| [ECM-receptor interaction](https://david.ncifcrf.gov/kegg.jsp?path=mmu04512$ECM-receptor%20interaction&termId=450049263&source=kegg) | 27 | 5.40E-10 |
| [Focal adhesion](https://david.ncifcrf.gov/kegg.jsp?path=mmu04510$Focal%20adhesion&termId=450049262&source=kegg) | 43 | 2.60E-09 |
| [Pathways in cancer](https://david.ncifcrf.gov/kegg.jsp?path=mmu05200$Pathways%20in%20cancer&termId=450049305&source=kegg) | 54 | 2.40E-07 |
| Small cell lung cancer | 20 | 3.10E-05 |
| [Cell cycle](https://david.ncifcrf.gov/kegg.jsp?path=mmu04110$Cell%20cycle&termId=450049243&source=kegg) | 23 | 4.70E-04 |
| [Axon guidance](https://david.ncifcrf.gov/kegg.jsp?path=mmu04360$Axon%20guidance&termId=450049260&source=kegg) | 23 | 6.60E-04 |
| [Regulation of actin cytoskeleton](https://david.ncifcrf.gov/kegg.jsp?path=mmu04810$Regulation%20of%20actin%20cytoskeleton&termId=450049290&source=kegg) | 32 | 1.10E-03 |
| [Renal cell carcinoma](https://david.ncifcrf.gov/kegg.jsp?path=mmu05211$Renal%20cell%20carcinoma&termId=450049307&source=kegg) | 15 | 1.10E-03 |
| [Bladder cancer](https://david.ncifcrf.gov/kegg.jsp?path=mmu05219$Bladder%20cancer&termId=450049315&source=kegg) | 11 | 1.40E-03 |
| [Wnt signaling pathway](https://david.ncifcrf.gov/kegg.jsp?path=mmu04310$Wnt%20signaling%20pathway&termId=450049255&source=kegg) | 22 | 7.60E-03 |
| [Pancreatic cancer](https://david.ncifcrf.gov/kegg.jsp?path=mmu05212$Pancreatic%20cancer&termId=450049308&source=kegg) | 13 | 1.10E-02 |
| [Adherens junction](https://david.ncifcrf.gov/kegg.jsp?path=mmu04520$Adherens%20junction&termId=450049265&source=kegg) | 13 | 1.70E-02 |
| [Galactose metabolism](https://david.ncifcrf.gov/kegg.jsp?path=mmu00052$Galactose%20metabolism&termId=450049139&source=kegg) | 7 | 1.80E-02 |
| [Heparan sulfate biosynthesis](https://david.ncifcrf.gov/kegg.jsp?path=mmu00534$Heparan%20sulfate%20biosynthesis&termId=450049183&source=kegg) | 7 | 1.80E-02 |
| [Tight junction](https://david.ncifcrf.gov/kegg.jsp?path=mmu04530$Tight%20junction&termId=450049266&source=kegg) | 19 | 2.20E-02 |
| [Amino sugar and nucleotide sugar metabolism](https://david.ncifcrf.gov/kegg.jsp?path=mmu00520$Amino%20sugar%20and%20nucleotide%20sugar%20metabolism&termId=450049179&source=kegg) | 9 | 2.20E-02 |
| [Melanoma](https://david.ncifcrf.gov/kegg.jsp?path=mmu05218$Melanoma&termId=450049314&source=kegg) | 12 | 2.50E-02 |
| [Non-small cell lung cancer](https://david.ncifcrf.gov/kegg.jsp?path=mmu05223$Non-small%20cell%20lung%20cancer&termId=450049319&source=kegg) | 10 | 2.70E-02 |
| [Prostate cancer](https://david.ncifcrf.gov/kegg.jsp?path=mmu05215$Prostate%20cancer&termId=450049311&source=kegg) | 14 | 2.70E-02 |
| [Basal cell carcinoma](https://david.ncifcrf.gov/kegg.jsp?path=mmu05217$Basal%20cell%20carcinoma&termId=450049313&source=kegg) | 10 | 3.00E-02 |
| [Proteasome](https://david.ncifcrf.gov/kegg.jsp?path=mmu03050$Proteasome&termId=450049228&source=kegg) | 9 | 3.20E-02 |

**Supplementary Table 3: Mice genotyping primers.**

| **Gene** | **Sequence** |
| --- | --- |
| *Wwox WT F* | 5” AGGGACGGCTGGGTGTACTA “3 |
| *Wwox WT R* | 5” CAACCTACTAGCCTCTCCAC”3 |
| *Wwox cKO F* | 5” AGGGACGGCTGGGTGTACTA”3 |
| *Wwox cKO R* | 5” ACCAAAGAACGGAGCCGGTT”3 |
| *Trp53 F* | 5” CACAAAAAACAGGTTAAACCCAG”3 |
| *Trp53 R* | 5” AGCACATAGGAGGCAGAGAC”3 |
| *Cre F* | 5” ATGTCCAATTTACTGACCGTACACC”3 |
| *Cre R* | 5” CGCCTGAAGATATAGAAGATAATCG”3 |

**Supplementary Table 4: qPCR primers for cDNA.**

| **Gene** | **Sequence** |
| --- | --- |
| *Wwox F* | 5” TCACACTGAGGAGAAGACCCA”3 |
| *Wwox R* | 5” CCTATTCCCGAATTTGCTCCA”3 |
| *Hprt F* | 5” TCAGTCAACGGGGGACATAAA”3 |
| *Hprt R* | 5” GGGGCTGTACTGCTTAACCAG”3 |
| *Trp53 F* | 5” TGAAACGCCGACCTATCCTTA”3 |
| *Trp53 R* | 5” GGCACAACACGAACCTCAAA”3 |
| *Cdkn1a (p21) F* | 5” TCCACAGGCATATCCAGACA”3 |
| *Cdkn1a (p21) R* | 5” AGACAACGGCACACTTTGCT”3 |
| *HPRT F* | 5” TGACACTGGCAAAACAATGCA”3 |
| *HPRT R* | 5” GGTCCTTTTCACCAGCAAGCT”3 |
| *TP53 F* | 5”GAGTATTTGGATGACAGAAACACTTT”3 |
| *TP53 R* | 5” CCAGTGTGATGATGGTGAGG”3 |
| *CDKN1A (P21) F* | 5” CTGGAGACTCTCAGGGTCGAA”3 |
| *CDKN1A (P21) R* | 5” GGCGTTTGGAGTGGTAGAAATCT”3 |
| *PUMA F* | 5” ACGACCTCAACGCACAGTACGA”3 |
| *PUMA R* | 5” GTAAGGGCAGGAGTCCCATGATGA”3 |
| *ESR1 F* | 5” CAAGTGGCTTTGGTCCGTC”3 |
| *ESR1 R* | 5” CTGTTGAATCAAACTCAATGGGC”3 |
| *PGR F* | 5” CAAAACCTGACACCTCCAGTT”3 |
| *PGR R* | 5” GCCACATGGTAAGGCATAATGA”3 |

**Supplementary Table 5: qPCR primers for genomic DNA.**

| ***Gene*** | **Sequence** |
| --- | --- |
| *Hprt F* | 5” CCAGGTTGGTGTGGAAGTTT”3 |
| *Hprt R* | 5” CATCTCGAGCAAGACGTTCA”3 |
| *Trp53 F* | 5” TCACCCTCAAGGTACCAAGG”3 |
| *Trp53 R* | 5” GAGAACCACTGTCGGAGGAG“3 |
| *Wrap53 F* | 5” CTGTCGTTCTCACAGGTCCA”3 |
| *Wrap53 R* | 5” AGAAACGAATCTCCCCGAGT”3 |
| *Shbg F* | 5” ACTCAGGCAGAAGGAAGCAG”3 |
| *Shbg R* | 5” TTGCGGAAATTCAGACTGTG”3 |
| *Sat2 F* | 5” CCTGCAGTTATCCTCCGTGT”3 |
| *Sat2 R* | 5” CCGGTCCATGTGCTATAGATG”3 |
| *Atp1b2 F* | 5” CTGCCATTAGCAGGGAACAT”3 |
| *Atp1b2 R* | 5” GCCTGCTGTTCCTAACATCC”3 |
| *Fxr2 F* | 5” ACTCAGGGCTCTGTTTCAGC”3 |
| *Fxr2 R* | 5” CTTGTAGAAGGCCCCGTTG”3 |

**Supplementary Table 6: sgRNA sequences.**

| sgRNA | Sequence 5”—“3 | targeting |
| --- | --- | --- |
| sgRNA 1 | CACCGCATGGCAGCGCTGCGCTACG | Exon 1 |
| sgRNA 2 | CACCGCTCCAGGGCCGGGATTTCAC | Exon 4 |
| sgRNA 3 | CACCGTGGTCACTGGAGCTAATTC | Exon 4 |

**Supplementary Table 7: PAM50 values.**

|  | **Basal** | **Her2** | **LumA** | **LumB** | **Normal** |
| --- | --- | --- | --- | --- | --- |
| **P53_KO_1** | 0.17764011 | -0.09283004 | -0.29808385 | -0.04336551 | -0.07083513 |
| **P53_KO_2** | 0.12957138 | -0.02650979 | -0.38237484 | 0.15291152 | -0.20842838 |
| **WWOX_KO_T_1** | 0.08701289 | -0.02415424 | -0.18924824 | -0.09431199 | -0.02031261 |
| **WWOX_KO_T_2** | 0.19962064 | 0.0209849 | -0.29205983 | -0.077841 | -0.02247353 |
| **WWOX_KO_T_3** | 0.41752701 | -0.03087635 | -0.39543818 | -0.20729892 | 0.05286915 |
| **WWOX_KO_T_4** | 0.28461668 | -0.17532234 | -0.2766453 | -0.19102499 | 0.07073399 |

The values represent the Spearman correlations between the expression values of the PAM50 genes in each tumor and those of the subtype centroids (based on Parker et al. 2009).
